# Delayed response to environmental conditions and infra-seasonal dynamics of the short-beaked common dolphin distribution

**DOI:** 10.1098/rsos.220379

**Published:** 2022-11-30

**Authors:** C. Lambert, M. Authier, A. Blanchard, G. Dorémus, S. Laran, O. Van Canneyt, J. Spitz

**Affiliations:** ^1^ Observatoire Pelagis UAR 3462 CNRS-LRUniv, 5 allée de l’Océan, La Rochelle 17000, France; ^2^ Centre d’Etudes Biologiques de Chizé UMR 7372 CNRS-LRUniv, 405 Rte de Prissé la Charrière, Villiers-en-bois 79360, France; ^3^ Littoral ENvironnement et Sociétés UMR 7266 CNRs-LRUniv, 2 Rue Olympe de Gouge, La Rochelle 17000, France

**Keywords:** winter, Bay of Biscay, simulation, temporal lag, dynamic species distribution modelling

## Abstract

Cetaceans adjust their distribution and abundance to encountered conditions across years and seasons, but we poorly understand such small-scale changes for many species, especially in winter. Crucial challenges confront some populations during this season, such as the high levels of fisheries-induced mortality faced by the common dolphin (*Delphinus delphis*) in the Northeast Atlantic shelves. For such species, understanding the winter fine-scale dynamics is crucial. We aimed to identify the dolphin distribution drivers during the winters of 2020 and 2021, with a focus on determining the lag between changes in oceanographic conditions and dolphin distribution. The changes were related to temporal delays specific to the nature and cascading effects that oceanographic processes had on the trophic chain. By determining the most important conditions and lags to dolphin distributions, we shed light on the poorly understood intrusions of dolphins within coastal waters during winter: they displayed a strong preference for the coastal-shelf waters front and extensively followed its spatial variations, with their overall densities increasing over the period and peaking in March–April. The results presented here provide invaluable information on the winter distribution dynamics and should inform management decisions to help reduce the unsustainable mortalities of this species in the by-catch of fisheries.

## 1. Introduction

Mobile marine species live in a highly dynamic environment, in both space and time [1–3]. Cetaceans in particular can move over large distances within short periods of time according to the variations they perceive in their environment [4,5], as well as in accordance with social and individual knowledge and experience of seasonal patterns in habitat fluctuations [6,7]. Cetaceans respond to perceived environmental changes either directly or indirectly [3–5]. They react directly to physically perceivable changes in their environment, preferring environmental conditions that best suit their physiology (temperature, salinity, currents, etc. [8]), but also conditions providing clues as to where foraging resources are located, where they are abundant, and how accessible they are (gradients of temperature, salinity or currents, euphotic depths, mixed layer depth, etc. [3,5]). Conversely, indirect responses occur when individuals react to downstream effects of environmental variability through trophic cascades (e.g. blue whales have been shown to aggregate at an upwelling two weeks after a wind event triggered its formation [9]). Consequently, changes in distribution can lag behind the environmental stimuli, with longer lags for indirect responses than direct responses.

Fluctuations in cetacean distribution, abundance and habitat preferences may be observed across years [10,11], as well as across seasons [12–15], depending on the spatio-temporal scales of relevant processes [16]. There is some evidence of short time-scale movements [17], but poor understanding of the relationship between such infra-seasonal movements of populations and environmental conditions [18,19], inasmuch as the dynamics of spatial distributions at this temporal scale are at best poorly documented for most top predators. This is mostly due to the paucity of information about the whereabouts of top pedators, and in particular cetaceans, at fine scale, both spatially and temporally. Inferring spatial dynamics at such fine temporal scale (i.e. infra-seasonal) requires surveys repeated at a sufficiently high frequency (complete coverage of the study area at least once a month). Due to logistics and economic constraints, such high frequency surveys can only be achieved at intermediate or small spatial scales, and in regions close to shores. This lack of information is even more acute during the winter season, when poor observation conditions and harsh weather considerably limit the amount of time spent at sea. As such, winter surveys are limited in number and extent, and so is our understanding of the dynamics of cetacean spatial distributions during this season.

The short-beaked common dolphin (*Delphinus delphis*) is one of the most abundant cetacean species in the world, yet its ecology and spatial distributions are still poorly understood for most of its range [20]. In spring and summer, the species is pelagic and associated with oceanographic features enhancing productivity of an otherwise oligotrophic water column [21], such as shelf edges and tidal fronts [15,22–24]. Intrusions into coastal waters during the winter season seem frequent in some regions (Gulf of Maine [25]; Bay of Biscay (BoB), English Channel and Celtic Sea [26–28]; Hauraki Gulf [14]; Madeira [29]), but the drivers behind this pattern are not known. Overall, we still lack a clear understanding of the dynamics of the common dolphin distribution across and within seasons at fine and large scales.

In the Northeast Atlantic, common dolphins in the BoB (mid-latitude European shelf) in winter also face a particularly high level of fisheries-induced mortality [30,31]. The by-catches in fishing gears are the most critical threat to the species in the area, with common dolphins experiencing high mortality in late winter (February–March) through several peaks of one or two weeks each winter [32]. This inter-annual fluctuation suggests by-catch risk varies over space and time, and potentially with environmental conditions [33]. Yet, since we lack knowledge of common dolphin distribution during the winter, we still do not fully understand the mechanisms driving those by-catch peaks, nor why they have intensified in recent years (i.e. whether it is due to an increase in abundance of dolphins, fisheries or both; or due to increased interactions between dolphins and fisheries). Understanding and describing the fine-scale spatial dynamics of common dolphin distribution and abundance during the winter is therefore a crucial step to the implementation of informed by-catch mitigation measures.

Building from aerial surveys unprecedented in their temporal scale, deployed during the winters of 2020 and 2021 (January–March) in the BoB, we investigated how common dolphin distribution was related to the temporal variations of environmental conditions during the winter on a daily basis. More specifically, we tested: (i) the extent to which common dolphin distribution lags behind changes in environmental conditions; (ii) the variables and lag time periods that best describe common dolphin distributional variability during the winter; and (iii) the quality of the predicted daily variability in distribution by these lag times and covariates.

To answer those questions, we first estimated the abundance of common dolphins within the study area with conventional distance sampling (CDS) methods for every survey date. We then tested which lag common dolphin distribution was better related to for a set of 31 environmental conditions, and established the species habitat preferences (i.e. preferential usage of environmental conditions relative to their availability [34]) for the winter period with generalized additive models (GAMs). We used these preferences to predict the distribution of common dolphins for every day throughout the winter season, from the start of January to mid-April, for the two winters. Finally, we set up a validation procedure based on simulations (commonly used in forecast model assessment) to assess the predictive quality of the models against two challenger models built up, assuming no variations in distributions across the winter.

## 2. Methods

### 2.1. Study area

**Figure 1.**
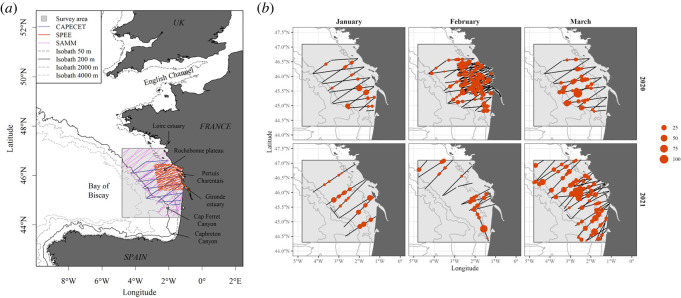
(*a*) Study area, detailing the three survey sampling schemes (CAPECET in blue, SPEE in red, SAMM in pink), the isobaths (50, 200 (shelf edge), 2000, 4000 m) and the geographical features mentioned in the text. (*b*) Survey design and common dolphin sightings by month from January to March, contrasting the winters of 2020 and 2021. The size of points is proportional to the number of individuals recorded by sighting. Land is displayed in plain grey, and isobaths (50, 20, 2000, 4000 m) are shown with grey lines.

The BoB (figure 1) is one of the westernmost bights of the western European shelves and is characterized by a variable continental shelf margin, ranging from broad in its northern part (180 km) to narrow in its southern part (20 km, Capbreton Canyon and Iberian coasts [35]). The oceanic part of the BoB is 2000–5000 m deep. The general circulation originates from the north-Atlantic gyre and flows from north to south [35,36]. A poleward slope current also circulates from south to north along the shelf break and is stronger during the winter. In the southern part, the interaction between this circulation, the topography and the wind-field dynamics can result in meso-scale eddies that are relatively slow and have long persistence (from several weeks to 1 year [37,38]). These eddies, which influence the exchanges of water across the shelf edge [39], intrude the southern edge of our study area. Over the shelf, currents are mainly driven by winds, tides or freshwater inputs. Tidal currents predominate in the northern BoB, while wind-driven currents predominate elsewhere, generating an important spatio-temporal variability of local currents [35]. The BoB ecosystem is largely influenced by freshwater inputs from large estuaries generating large river plumes (mainly the Loire and Gironde estuaries [40]) and from the Pertuis Charentais system. They induce a strong thermohaline stratification supporting productivity, even in winter if the radiance is sufficient, which is mainly restricted within the 50 m isobath [35]. The plume extension depends on the dominant current flow and the Gironde plume can mix with the Pertuis Charentais freshwater inputs when dragged northward. These general circulation patterns result in the BoB being characterized by strong frontal activities with important seasonal dynamics [41]: in winter (January–March), an important frontal regime is apparent along the 30–100 m isobaths, mostly resulting from interactions between tidal forcing, wind and river plumes. This front displays spatial variations and intensity fluctuations depending on current dynamics and freshwater inputs, and supports a large part of the winter productivity and biodiversity in the area [41].

### 2.2. Aerial surveys

This work was based on three independent aerial surveys of various extents covering the central part of the BoB shelf, from south of the Loire estuary to the south Gironde coast: the CAPECET, SPEE and SAMM surveys. The CAPECET survey extended from the coast to the 2000 m isobath (figure 1*a*). Four sessions were conducted between January and March 2020, for a total sample of 6653 km (table 1). The SPEE survey was a medium-term survey, with four sessions a year between 2019 and 2021. For the purpose of this work, we focused on the winter sessions, conducted in February 2020 (session 5, totalling 3583 km) and February–March 2021 (session 9, 1738 km; table 1). The SAMM survey had the largest extent of the three surveys, covering the entire BoB and the English Channel, and was conducted from January to March 2021. For the purpose of this work, we only considered the effort flown within our study area, totalling 4211 km (table 1).

**Table 1.** Summary of the total effort (km) flown by survey session, the total effort flown in good observation conditions (with Beaufort sea state lower than or equal to 3, and medium to excellent subjective conditions), the total number of individuals and the total number of sightings (both summarized on good observation condition effort).

date	survey (session)	total effort	effort in good conditions	*N* individuals	*N* sightings
11, 24, 26 Jan 2020	CAPECET (1)	1748	1410	141	16
5–6 Feb 2020	CAPECET (2)	1709	1098	138	32
12 Feb 2020	SPEE (5)	3583	3565	743	135
Feb 2020, 23.24	CAPECET (3)	1422	785	45	7
Mar 2020, 12.13	CAPECET (4)	1774	1768	690	86
total 2020		10 236	8626	1757	276
11, 18, 25 Jan 2021; 5, 13, 25 Feb 2021; 3, 7, 9, 16, 25 Mar 2021	SAMM	4211	4096	1208	135
10, 25 Feb 2021; 23–25 Mar 2021	SPEE (9)	1738	1660	735	95
total 2021		5949	5756	1943	230

All surveys followed a standard, validated multi-species observation protocol [42,43], with a zig-zag layout designed to maximize coverage of the study area in each session ([44], figure 1). An observer team of three experienced scientists with similar detection abilities, who were used to working together, conducted surveys on-board a highwing double-engine aircraft (Partenavia P68 or Britten Norman II Islander) equipped with bubble windows. The three observers switched observation posts at regular intervals to reduce fatigue. The aircraft flew at a constant speed of 90 knots (167 km h^−1^) and height of 600 feet (183 m). Observers visually recorded all environmental conditions impacting the detection of animals, following standardized, calibrated scales, specifically: sea conditions measured on the Beaufort scale (hereafter ‘sea state’), turbidity, swell intensity, cloud cover, sky glint, glare severity, glare orientation and the ‘subjective condition’ which integrates all the above factors and is defined as the probability of an observer detecting a small cetacean at the surface [43]. All flight information and sightings were collected using SAMMOA 1.0.4 software [45]. The cetacean sightings were recorded following a line-transect protocol, with all animal detections duly recorded alongside their location and the detection angle, as well as the behaviour, the swimming direction, an estimation of the number of animals present during each sighting event and the presence of juveniles.

### 2.3. Detection functions and density estimations

The effort was divided into 5 km segments of homogeneous detection conditions using the pelaSIG plug-in in QGIS [46], and further prepared for distance analysis using the in-development pelaCDS and pelaDSM packages in R (v. 4.0.4; [47,48]). Only segments with good conditions were used for the upcoming analyses: we only retained data with a sea state lower than or equal to 3, and medium to excellent subjective conditions. This resulted in: 8626 km of effort (76% of CAPECET, 99% of SPEE) and 1757 sighted common dolphins for the winter 2020 (table 1); and 5756 km of effort (97% of SAMM, 96% of SPEE) and 1943 sighted individuals for the winter 2021 (figure 1*b*, table 1).

A CDS procedure was applied to derive density estimates and effective strip half-width (ESW), using the pelaCDS package [48]. We implemented CDS models, testing the hazard and half-normal distributions on data right truncated at 400 m, to remove extreme distances (visual assessment [49]). Model fit was visually assessed and model performance compared with AIC. The model with the smallest AIC was used to estimate the survey-specific ESWs and the density of common dolphins during each survey session and day.

### 2.4. Environmental conditions

We aimed to describe the changes in common dolphin distribution at a fine spatio-temporal resolution, focusing on the short-term, contemporaneous oceanographic processes known to drive the spatial patterns of the species [16]. As such, we used oceanographic variables available at fine scale, both temporally (daily) and spatially (0.083°).

The environmental conditions summarizing the BoB ecosystem were extracted for 1 December to 13 April from the Marine Copernicus platform (http://marine.copernicus.eu), using the Iberian-Biscay-Irish physical and biogeochemical forecast products, at the daily resolution. These products include a wide array of physical and biogeochemical variables at small spatial resolution (0.083°). We used the daily mean values at the surface layer of sea surface temperature (Temp; °C), salinity (Salinity), sea surface height (SSH; m), mixed layer depth (MLD; m), dissolved inorganic carbon (DissIC; mol m^−3^; a proxy of the water concentration in inorganic matter), surface partial pressure (SPCO2; Pa), euphotic depth (ZEu; m), chlorophyl surface concentration (Chl; mg m^−3^), net primary production (NPP; mg m^−3^ d^−1^) and phytoplankton concentration (Phyto; mmol m^−3^).

We also derived the current speed (CurrentSpeed; m s^−1^) from the northward and eastward seawater velocities available in the above-mentioned products (computed as $\sqrt{}(U^{2}+V^{2})$, where *V* is the northward velocity and *U* is the eastward velocity), and the eddy kinetic energy (EKE; cm^2^ s^−2^; computed as 0.5 × (*U*^2^ + *V*^2^)). Daily gradient (grad) maps were calculated for Temp, MLD, SSH, Salinity, NPP and Chl using the detectFronts functions (from the grec package [50])—large gradient values are indicative of frontal areas. In addition to the gradient maps, we derived daily maps of distance to fronts for Temp, MLD, NPP and Chl.

Finally, we considered the bathymetry (GEBCO 2008 database http://www.gebco.net) and two long-term variables: the winter-averaged distance to front of Temp, MLD, NPP and Chl (December–April averages in 2020 and 2021, separately), and the frontal persistence of front over the winter (the number of days each cell includes a front across the study period), also for Temp, MLD, NPP and Chl.

### 2.5. Temporal scale of dolphin response to environmental conditions

**Figure 2.**
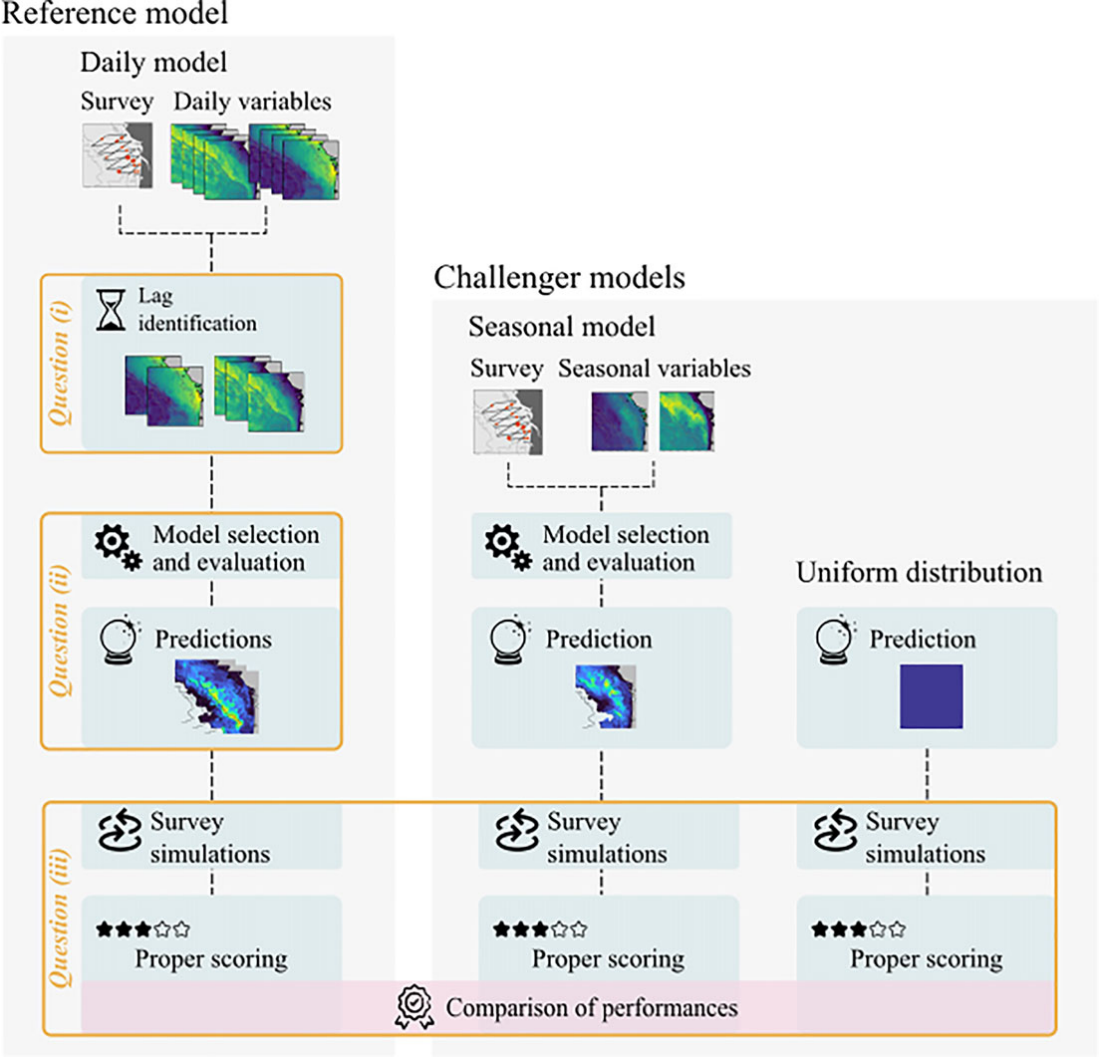
Workflow of analyses used in the present study, contrasting the three main objectives (orange boxes) and the three spatial distribution models implemented. The reference model was a temporally dynamic model at the daily scale, providing daily predictions of common dolphin distribution. This model was used to answer the two first objectives of the work. The temporally static seasonal model was computed from seasonal-scale variables to construct winter-scale predictions. This model was used alongside a uniform spatial distribution as challenger models to answer objective (iii): to assess the relative performance of the reference model to reliably predict common dolphin distributions. This performance assessment was conducted by means of proper scoring the number of common dolphins sighted during simulated surveys built from predicted distributions against the true observed values.

Mobile marine animals adjust their spatial patterns depending on the environmental conditions they perceive and experience, and the changes in distribution can lag behind the environmental changes depending on the nature of the latter [3–5,9]. As such, the first objective of the study was to quantify such a lag in our study area (figure 2). Phytoplankton communities are known to respond environmental forcing factors in 0 to 5 days, depending on systems and factors [51]. With trophic cascades being sequential in nature, the response of upper levels would scale up the trophic chain within days and weeks [9], so that we can expect the lags between dolphin distribution and oceanographic processes to be in that range of scales.

The dynamic environmental conditions we considered can be sorted into five different categories according to the type of processes they are related to (some can be included in several categories): physiological effects (thermo- or osmoregulation [8]), water column enrichment, prey availability, prey accumulation and prey accessibility (table 2). These environmental changes should be reflected in common dolphin distribution with time lags directly dependent upon the distance between the process itself and the dolphin response. We also tested the effect of bathymetry and longer-term features, such as the winter-averaged distance to fronts of Temp, MLD, Chl and NPP, as well their persistence over the winter (the proportion of days a front is present in a cell).

**Table 2.** Categorisation of dynamic environmental conditions depending on the expected common dolphin response to the processes they are proxies of, with the corresponding expected time lag (Temp, sea surface temperature; EKE, eddy kinetic energy; MLD, mixed layer depth; DissIC, dissolved inorganic carbon (a proxy of the water concentration in inorganic matter); Chl, chlorophyl surface concentration; NPP, net primary production; Phyto, phytoplankton concentration; ZEu, euphotic depth; SSH, sea surface height). gradTemp, gradMLD, gradSSH, gradSal, gradNPP and gradChl are gradients of Temp, MLD, SSH, Salinity, NPP and Chl, respectively (proxies of frontal areas).

category	process	variables	expected lag
physiological effect	thermoregulation, osmoregulation, energetic balance	Temp, Salinity, CurrentSpeed	short
water column enrichment	water mixing and nutrient influx, ultimately boosting productivity	EKE, Temp, gradSSH, gradTemp, gradMLD, gradSal, DissIC	long lag, corresponding to the time the effect reverberates/scales up to the upper trophic chain
prey availability	proxies of trophic chain activity and prey concentration	Chl, NPP, Phyto	long lag but a bit shorter than for water column enrichment variables, as they are closer to common dolphins in the reaction chain and can be directly perceived
prey accumulation	proxies of oceanographic processes favouring prey accumulation in frontal areas or eddy edges	SSH, CurrentSpeed, EKE, gradMLD, gradSSH, gradTemp, gradChl, gradNPP, gradSal	short lag
prey accessibility	vertical accessibility	ZEu, MLD (proxies for the mixed layer depth)	short lag
	horizontal accessibility	CurrentSpeed, distance to fronts (of Temp, MLD, Chl, NPP)	

We associated sampled segments with the values of environmental conditions underlying their centroids using the extract function (from the raster package [52]). Static and long-term variables were extracted as is, but dynamic variables were incorporated with seven different lags: none, 1, 2, 4, 7, 10 and 30 days prior to the day each segment was sampled. Those lags were chosen to incorporate immediate response (no lag, 1 d lag), delayed response with different integration time (2–10 d lag) and longer-term response (30 d lag). Variables with outliers were saturated to facilitate model fitting without discarding data: values below the 1% or 2%, and/or above the 98% or 99%, quantiles were set to this quantile value where necessary. The threshold quantile value was chosen based on visual inspection of data.

We conducted a model selection procedure to compare the performances of each time lag for every dynamic variable. GAMs with one covariate were fitted with the gam function (from the mgcv package [53]), using the number of individuals sighted per segment as the response variable, assuming a Tweedie distribution, and using the REML estimation method on thin plate regression splines with a maximum complexity level of 4. The sampled area associated with each effort segment was used as an offset (2 × ESW × segment length). GAMs were chosen in order to benefit from their flexibility: penalized smooths permit the identification of a relationship between the response variable and a predictor without *a priori* knowledge about its shape, which was the case here.

For each variable, we fitted one model per lag (0, 1, 2, 4, 7, 10, 30 days) plus a null model (only the intercept), and retrieved the AIC and the explained deviances of the eight models. These models were sorted by AIC values. Next, the Delta AIC, Akaike weights and relative likelihood of each model were computed using the akaike.weights() function (from the qpcR package [54]). For each variable, the lags achieving a Delta AIC lower than 2 were considered to be the most predictive of common dolphin distribution. Results were double-checked using the root mean square errors (RMSE) of models, also computed using the qpcR package. The non-dynamic variables were tested using the same approach, but against the null model only. Finally, we visually inspected the estimated splines from each model.

### 2.6. Model selection

Once we had identified, for every dynamic variable, which lag the common dolphin distribution was better related to, we performed a habitat model selection procedure, including the variables identified in the previous steps (figure 2). For each variable, only lags with Delta AIC lower than 2 were considered. In addition, we discarded all variables whose best performing lag had an Akaike weight lower than 50% and all variables which did not perform better than the null model. This pre-selection resulted in a total of 28 variables: Temp (10 days), Salinity (10 days), Chl (7 days), NPP (10 days), Phyto (10 days), EKE (10 days, 4 days), ZEu (7 days), MLD (10 days), DissIC (10 days), SPCO2 (10 days), gradMLD (10, 30, 2 days), gradSal (10 days), gradSSH (10 days), distance to MLD fronts (4 days, 30 days), distance to Temp fronts (30 days), distance to Chl fronts (2 days), distance to NPP fronts (4 days), average distance to Chl, NPP and MLD fronts, and persistence of Temp, Chl, NPP and MLD fronts.

The selection procedure tested every combination of up to four variables in the models, using a restrictive threshold in handling multicollinearity issues by removing all combinations of variables with a correlation higher than 60% [55,56]. As before, the complexity level in splines was constrained to 4, GAMs were adjusted with the REML method with a Tweedie distribution and the sampled area associated with each effort segment was used as an offset (2 × ESW × segment length). For each tested model, we retrieved the AIC, and the residual and null deviances from which we computed the explained deviance. Models were sorted by AIC values. Next, the Delta AIC, Akaike weights and relative likelihood of each model were computed using the akaike.weights function in R. Potential best models were those with the largest Akaike weights. The response curves for the selected best model were plotted based on predicted values using the itsadug package [57].

The selection procedure identified two potential best models, sharing three out of four variables and with similar performances. To help identify the most informative fourth variable to be kept in the final best model, we determined the importance of each of the 28 variables by summing the Akaike weights of each model in which each variable was selected, and then ranking all variables. The final best model was constructed using the best-ranked candidate variable among the two as the fourth variable.

We predicted the daily distribution of common dolphin density in the study area from 1 January to 13 April for both winters, based on the selected best model, using the predict.gam function (mgcv [53]). We then computed the winter-scale mean and variance maps, and summarized the mean and variance of predicted density over the study area for each day of the time series. The same procedure was done with the prediction uncertainty.

We quantified the degree of extrapolation of each prediction grid compared to the environmental data used to fit the reference model. This procedure was conducted using the WhatIf package [58] and allowed us to assess the quality of the predictions [59].

### 2.7. Prediction reliability assessment

#### 2.7.1. Principle

We used a validation procedure that is commonly used in forecast model assessment to assess the reliability of predictive models [60,61]. It is based on simulations, and allows the reproduction and maintains the coherence of the sampling design behind the reference model. The rationale is to simulate alternative aerial surveys following the original survey tracks (segments) on an underlying species distribution that is generated with the distributions predicted by our model. In other words, the aim is to simulate a survey considering the predicted distribution is true and to compare the obtained dataset with what was actually observed; the closer the simulated values are to the observed ones, the more accurate the predicted distribution is. This procedure was used to simulate the surveys conducted during both winters (CAPECET and SPEE-5 in 2020; SAMM and SPEE-9 in 2021).

Prior to launching simulations, we need to use challenger models to generate common dolphin distributions, against which the performance of our reference model can be assessed (figure 2). To this aim, we considered two alternative models with a different underlying hypothesis regarding the spatio-temporal variations of common dolphin distribution during the winter: a uniform distribution where the dolphins are homogeneously distributed in the study area through both space and time; and a seasonal model where the dolphins are distributed following an environmentally driven pattern over the study area, but this pattern is fixed in time and does not change through the winter (unlike the daily reference model).

We expect the lowest performance for the uniform distribution. The comparison between the daily (reference) and seasonal models will provide supplementary information on the common dolphin winter ecology, in addition to assessing the reliability of our reference model.

#### 2.7.2. Challenger seasonal model

The seasonal (i.e. non-dynamic) model was built using the same environmental variables as previously (see §2.4) but averaged over the winter (means). We also included their standard deviations (sd) over the same period to account for their variations during the season. We implemented a model selection procedure similar to the one used to select the reference daily model, with data from the two winters used jointly to calibrate the model. Prediction maps were derived from this model for the winters of 2020 and 2021.

#### 2.7.3. Evaluation of prediction reliability

The simulations of aerial surveys and detection process were done at the segment scale and iterated 100 times. For each sample day, we converted the prediction map into an Inhomogeneous Poisson Point Process (IPPP), using the spatstat package [62]. This method generates a spatial point process with an intensity proportional to the predicted density, so that the simulated number of common dolphin positions per cell is proportional to the predicted density (individuals per km^2^).

For the daily model, the reference map was different according to the day the segment was sampled (daily predictions), while the reference map was the same for all segments sampled during the same winter for the seasonal model, and the same for all segments whatever the year for the uniform distribution. However, since the IPPP is stochastic, the distributions generated through every single simulation and for every segment were unique. We then discarded all points further than 700 m from the segment, using the st_is_within_distance function in the sf package [63], as this corresponds to the maximum distance to which common dolphins were sighted during our aerial surveys. This approach is conservative, since, in the CDS analysis, we discarded sightings further than 400 m away. The precise distance to the survey track was calculated for all remaining points (using the sf::st_distance function). A point was considered detected if its distance to the track line was shorter than or equal to the survey-specific ESW identified through CDS (see §2.3). The number of detected individuals for the segment was retrieved as the sum of detected points.

Following this procedure, we obtained, for each segment, 100 simulated values for the number of individuals sighted. Only segments sampled with good observation conditions were considered for the analysis (sea state less than or equal to 3, and medium to good subjective conditions), i.e. 2352 segments.

A proper scoring rule was used to compare the distribution of values obtained from the 100 simulations to the observed values, using the quantile decomposition of the continuous ranked probability score (CRPS; crps_sample function in the scoringRules package [60,61]). This method quantifies the distance of a punctual observation (here, the number of individuals obtained from the real survey) from a predicted distribution of this value obtained through simulation. We visually compared the density distributions of scores across the three models to assess the difference in performance (the lower the scores, the better the predictions from a model).

## 3. Results

### 3.1. Oceanographic conditions

In our study area, two water masses stand out (see electronic supplementary material at https://github.com/CLambert1/WinterBoBDd for day-to-day and season-averaged maps of environmental conditions). The first runs parallel to the coast, stretching from there to the 50 m isobath, and is under the influence of tidal mixing, river plumes and winds. Low temperature and low salinity are characteristics of this water mass, which sustains an important fraction of the phytoplankton biomass produced during the winter. The second water mass extends from the 50 m isobath towards the west, covering the entire shelf and beyond, with a deep MLD, warmer temperature and rather oligotrophic conditions.

An important MLD front occurs at the edge of those two water masses, more or less expanded and sinuous depending on the year. The coastal waters stretched out towards the shelf and were deeper than the 50 m isobath by the end of February, early March, probably through the effects of winds that dispersed the plumes towards the west. By the end of March, the MLD front reached and merged with the shelf edge front before disappearing in April. The late-winter front dispersion coincided with the establishment of spring conditions, with increases in productivity and concentrations of phytoplankton, probably triggered by the establishment of the nutrient-rich water stratification (as shown by the drop in MLD; see variable time series in electronic supplementary material at https://github.com/CLambert1/WinterBoBDd) combined with increased solar radiance.

### 3.2. Observed encounter rates, detection functions and density estimations

The daily encounter rates of common dolphins in the studied area did not display a particular trend over the season, ranging from 0.04 to 0.26 individuals per km in 2020 and from 0.00 to 0.43 in 2021, despite the rates being somewhat lower in January compared with February–March in 2020.

The best CDS model was fitted with the half-normal distribution. The ESW was estimated to be 0.17 km [0.15; 0.19] (lower and upper bound of the 95% confidence interval) for the CAPECET survey, 0.16 km [0.14; 0.17] for the SPEE-5 survey, 0.20 km [0.17; 0.23] for the SAMM survey and 0.18 [0.16; 0.21] for the SPEE-9 survey.

The density estimations were retrieved for each survey date. The densities followed the same pattern as the encounter rates. In 2020, the densities were lowest in January (from 0.08 ± 0.06 to 0.31 ± 0.14 individuals per km^2^) and highest in March (from 1.04 ± 0.37 to 1.27 ± 0.66 ind. per km^2^). In 2021, the densities decreased through January (from 0.92 ± 0.48 to 0.42 ± 0.37 ind. per km^2^), before increasing in February to reach a peak in late March (2.19 ± 1.93 ind. per km^2^). However, those trends are to be viewed with caution as the associated standard errors are wide (ranging from 0.04 to 1.93 ind. per km^2^).

### 3.3. Temporal scale of dolphin response to environmental conditions

**Figure 3.**
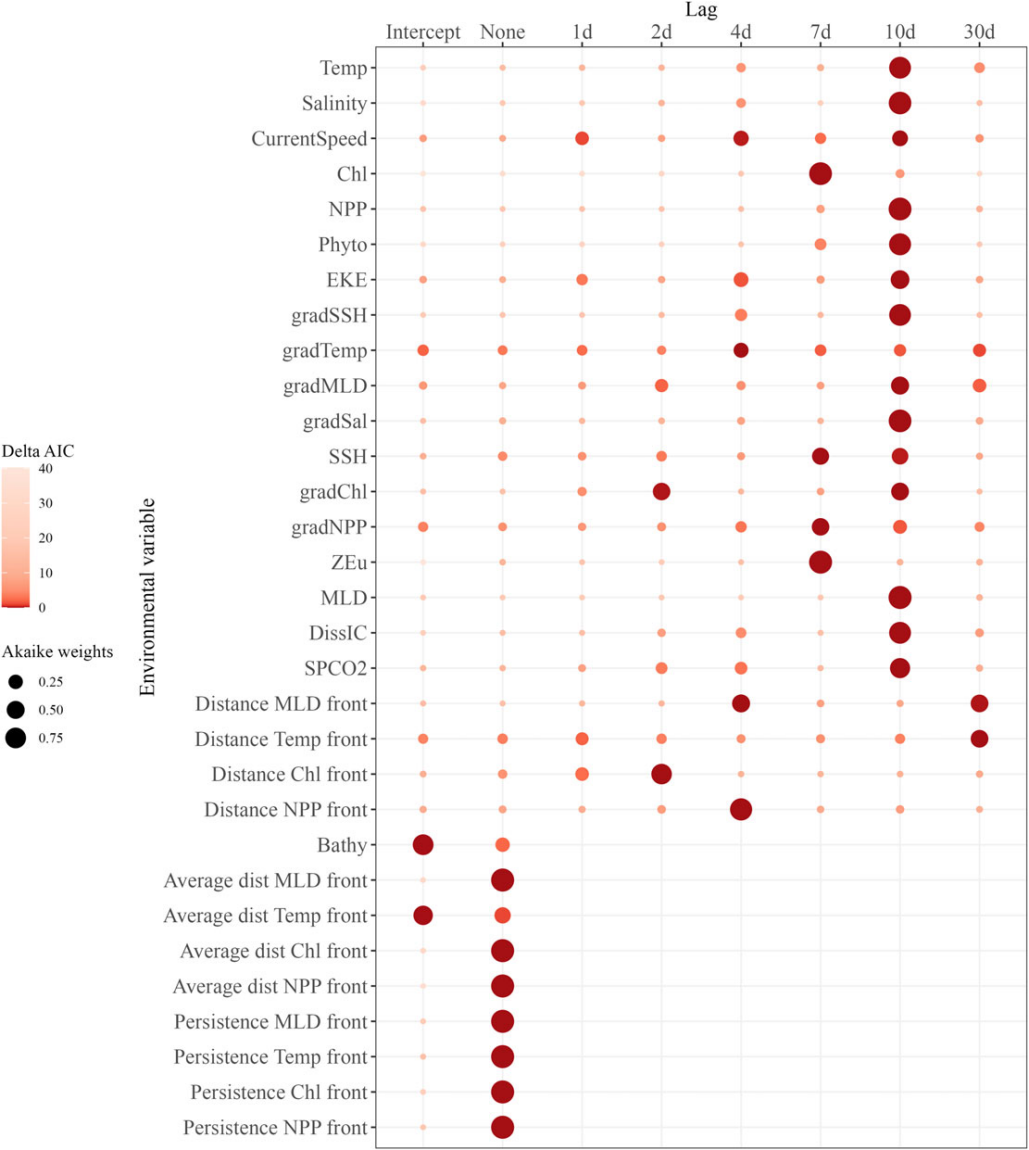
Akaike weights of each temporal lag by variable, as well as the intercept models. The colour is proportional to the weights characterizing each model and the text labels indicate the Delta AIC achieved by each one. Lags with a Delta AIC lower than 2 and the greatest Akaike weights were the best-performing lags. Temp, sea surface temperature; Chl, chlorophyl surface concentration; NPP, net primary production; Phyto, phytoplankton concentration; EKE, eddy kinetic energy; SSH, sea surface height; ZEu, euphotic depth; MLD, mixed layer depth; DissIC, dissolved inorganic carbon; SPCO2, surface partial pressure. gradTemp, gradMLD, gradSSH, gradSal, gradNPP and gradChl are gradients of Temp, MLD, SSH, Salinity, NPP and Chl, respectively (proxies of frontal areas).

The 10 d lag clearly stood out for many variables (figure 3), namely Temp, Salinity, NPP, Phyto, gradSSH, gradSal, MLD, DissIC and SPCO2, for which this lags obtained the strongest support (AIC weight ranging from 69% to 99%). Chl and ZEu worked best at a 7 d lag, achieving an AIC weight of 96% and 99%, respectively. The 4 d lag was best for the distance to NPP front (AIC weight of 90%), the 2 d lag was best for the distance to Chl front (73%) and the 30 d lag was best for the distance to Temp front (50%).

For the remaining dynamic variables, several lags stood out. This was the case for EKE, for which both 10 d and 4 d lags had a Delta AIC lower than 2, for AIC weight of 56% and 28%, respectively. For the gradMLD, the 10 d, 30 d and 2 d lags were the best (AIC weight of 51%, 21% and 19%, respectively), while for the distance to MLD front it was the 4 d and 30 d lags (AIC weight of 50% and 48%, respectively).

Several lags also stood out for gradChl, gradNPP, CurrentSpeed and SSH, with a Delta AIC lower than 2, but none achieved an AIC weight greater than 50%. The gradTemp was poorly informative at any lags, with none performing better than any other and none performing better than the intercept model for this particular variable. Those five variables were thus discarded from the analysis for the model selection procedure.

Two particular cases occurred with Bathy and average distance to Temp fronts, which were completely uninformative, with the intercept model achieving better results than the variable itself. *A contrario*, average distances to MLD, Chl and NPP fronts and persistence of Temp, Chl, NPP and MLD fronts were strongly informative compared with the null models, with an AIC weight >99% for all.

### 3.4. Winter distribution of common dolphins

#### 3.4.1. Reference model

Based on the 28 pre-selected variables, we tested a total of 8497 different models. Two models stood out as potentially the best model, with a Delta AIC lower than 2 (AIC weight of 16% and 15%; the third model had an AIC weight of 0.06). These two models had equivalent deviances: 12.2%. They both included Chl (7 d lag), and average distance to MLD fronts and distance to Chl front (2 d lag). The first model included as a third variable the persistence of Temp front, while the second incorporated MLD (10 d lag).

The AIC weight ranking of variables confirmed that Chl (7 d), and average distance to MLD fronts and distance to Chl fronts (2 d) had the greatest weights (99.5%, 81.6% and 57.8%, respectively). MLD (10 d) and persistence of Temp front came just after, with an AIC weight of 34.7% and 21.4%, respectively. We selected the model including Chl (7 d), average distance to MLD fronts and distance to Chl fronts (2 d), and MLD (10 d) as the best one.

**Figure 4.**
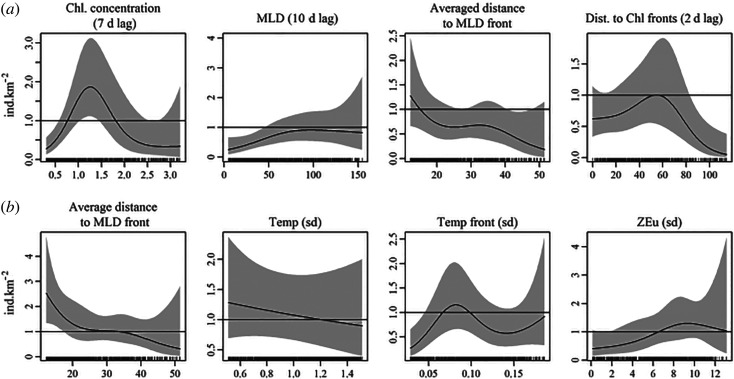
Estimated splines for the four environmental variables for (*a*) the daily model and (*b*) the seasonal model, provided in natural scale (number of individuals). Response curves are summed effects, i.e. they are predicted values and take into account the other variables (set to their mean) and the intercept.

The highest common dolphin densities were achieved with the intermediate Chl concentration (figure 4*a*), with a peak at 1.25 mg m^−3^, largest MLD (with a plateau at 80 m onwards) and lowest average distances to MLD front, and at a 50–60 km distance from Chl fronts.

#### 3.4.2. Spatial distribution

**Figure 5.**
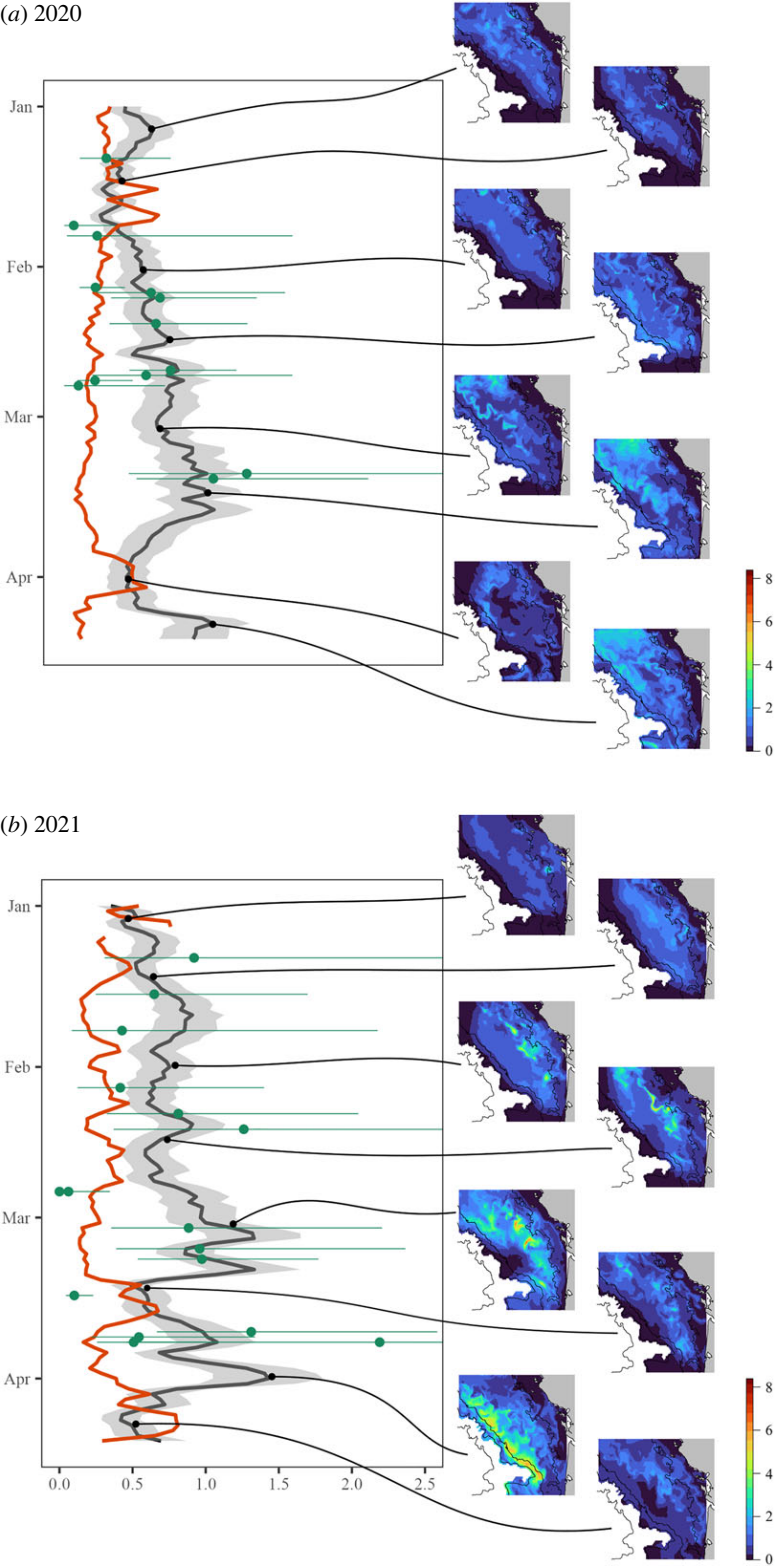
Time series of the predicted density of common dolphins (ind. per km^2^) from 1 January to 13 April for the (*a*) 2020 and (*b*) 2021 winters, with the daily mean predicted density in black (associated uncertainty in shaded grey) and the proportion of the daily prediction grids in extrapolation compared with the environmental data used to fit the model displayed in red. The densities estimated through CDS are shown in green (estimated over transects sampled on each day, not the whole survey area). The right panel displays predicted maps of distribution (ind. per km^2^) for selected days for illustration purposes (1 January, 15 January, 1 February, 15 February, 3 March, 15 March, 1 April, 10 April; for both years). The land is shown in grey and the isobaths with black lines. The complete day-to-day predictions can be found in the electronic supplementary material at https://github.com/CLambert1/WinterBoBDd.

The common dolphin distribution displayed important changes throughout the winter, following the winter dynamics of suitable environmental conditions (the day-to-day predictions can be found in the electronic supplementary material at https://github.com/CLambert1/WinterBoBDd, and figure 5 for some selected dates). In the study area, common dolphins were mostly present between the 50 m and 200 m isobaths during both winters. In 2020, common dolphins were distributed preferentially along the frontal systems north of 45.5°N, associated with the 50 m isobath in January–February before progressively sliding westward in March–April, when the highest densities were predicted north of 45.5°N. Few common dolphins were predicted close to the coast during this winter and none were predicted south of the Cap Ferret Canyon before mid-March. In 2021, a similar pattern occurred in January–March, with common dolphins predicted to be associated with the frontal system flourishing along the 50 m isobath, but more widespread than in 2020 and with the highest densities in the central part of the area (45−46°N; figure 5*b*) and around the Cap Ferret Canyon. In March–April, common dolphins were predicted throughout the shelf, along the front sliding westward, but also in the outer shelf and close to the Cap Ferret Canyon, with a burst of high densities along the 200 m isobath in early April. The extrapolation level was 27% on average (sd of 0.12) for the winter 2020 and 35% (sd of 0.17) for the winter 2021. However, this level was low within the survey area (see electronic supplementary material at https://github.com/CLambert1/WinterBoBDd) but greater outside, with some areas where extrapolation was systematic for all predicted days: along the northern coast and the Gironde estuary in 2020; and along the northern coast and in the central shelf in 2021.

#### 3.4.3. Time series of density

The density of common dolphins in the study area displayed a slight increase over the January–March period in 2020 (figure 5), from an average of 0.50 ind. km^−2^ in January to around 1 ind. km^−2^ in mid-March, steadily increasing in February except for a single drop to 0.50 ind. km^−2^ by 18 February. A sharp decrease to 0.50 ind. km^−2^ in early April followed this peak in March, before the density rose again to a peak of 1 ind. km^−2^ in mid-April.

In 2021, the time series was quite different, displaying more intense variations in densities (figure 5). Although the early days of January displayed similar densities to 2020, the densities for the rest of the month were higher, with a maximum of 0.91 ind. km^−2^. Densities fluctuated between 0.59 and 0.91 ind. km^−2^ in February, before rising to 1.19 ind. km^−2^ in early March. Strong oscillations occurred from March to April, with densities halving from around 1.00−1.43 ind. km^−2^ to 0.49, 0.68 and 0.42 ind. km^−2^.

The daily averaged extrapolation levels fluctuated throughout the period, with an opposite pattern to daily predicted densities (figure 5). In winter 2020, low levels of extrapolation were observed throughout the period (daily mean less than 40% overall), with the lowest values (9%) occurring during predicted peaks in common dolphin density. Conversely, extrapolation was highest when the predicted common dolphin density was the lowest (maximum extrapolation at 66% in late January). In winter 2021, the overall mean extrapolation was a bit higher than in 2021 (35% versus 27%), but a similar pattern occurred, with lows in extrapolation when common dolphin density peaked (minimum of 12%). Unlike in 2020, however, the extrapolation levels were important in early January and in April, with peaks at 76% and 81%, respectively.

### 3.5. Prediction reliability assessment

#### 3.5.1. Seasonal model

This model used the averaged distance to MLD fronts, the standard deviation of Temp, the standard deviation of gradTemp and the standard deviation of ZEu. Its deviance and AIC weight were 8.3% and 12%, respectively. The highest densities occurred with short distances to MLD front, lower standard deviation of Temp, intermediate values of gradTemp standard deviation (0.06–0.10) and large standard deviations of ZEu (6.5–13; figure 4*b*).

**Figure 6.**
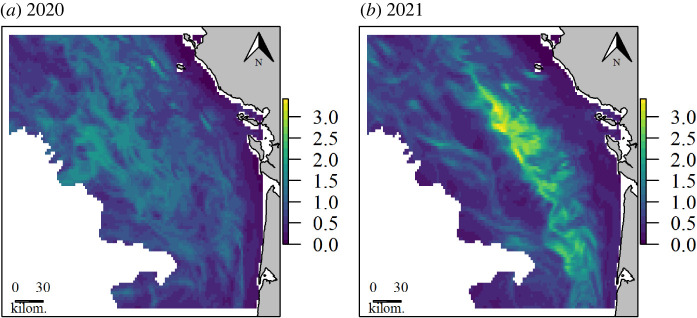
Predicted distribution of common dolphins from the seasonal model (ind. per km^2^) for the winters (*a*) 2020 and (*b*) 2021.

In 2020, the model predicted the common dolphins to be distributed throughout the study area, except in the coastal areas below the 50 m isobath. The highest densities were predicted in the western part of the area (figure 6). The extrapolation level was 40%. Extrapolations occurred mostly in the north-western part of the area and along the coast (see electronic supplementary material at https://github.com/CLambert1/WinterBoBDd). In 2021, the model also predicted the avoidance of the shallower parts of the study area, but also of the outer shelf. The highest densities were predicted along a strip running west of the 50 m isobath throughout the study area. The extrapolation level was lower than in 2020 (25%), with the extrapolation areas being scattered throughout the study area.

#### 3.5.2. Proper scoring

**Figure 7.**
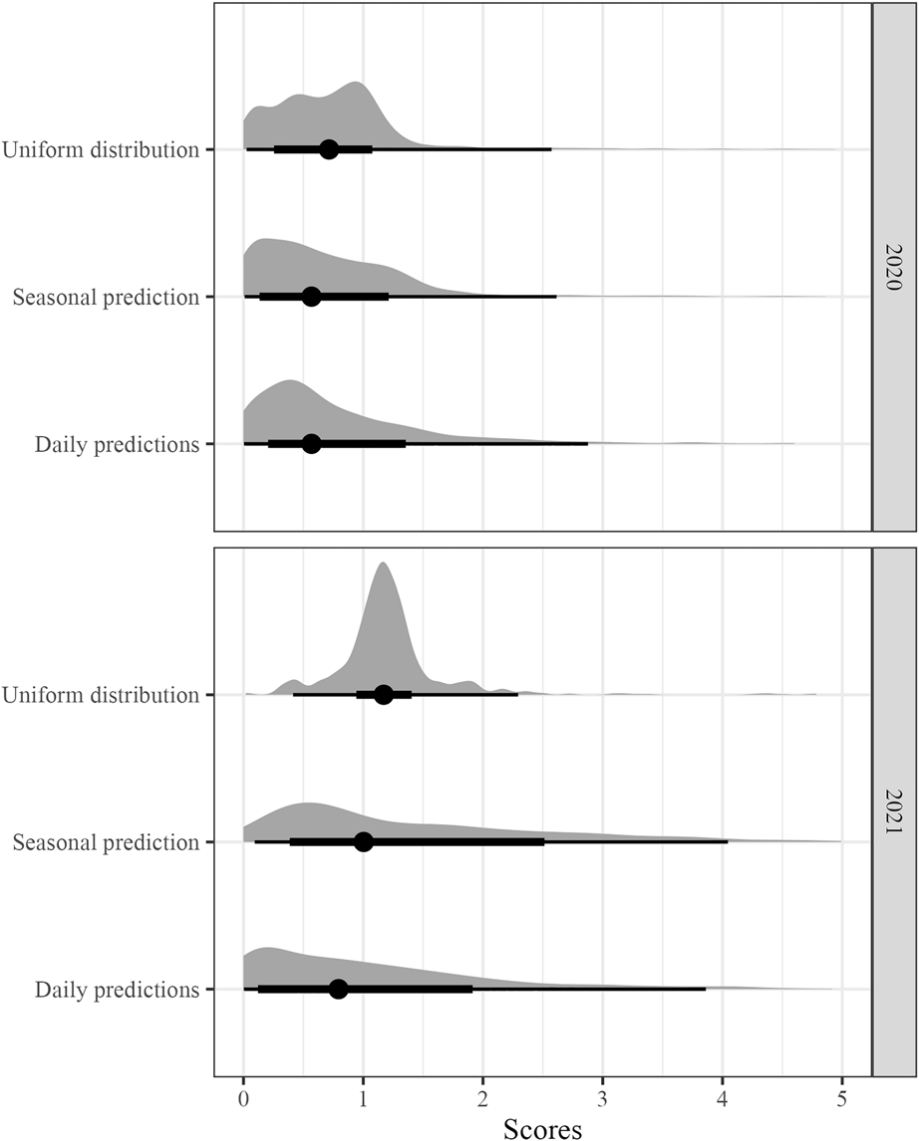
The density distributions of the scores obtained by the uniform distribution, the seasonal prediction and the daily predictions for the winters of 2020 (top) and 2021 (bottom). The *x*-axis is truncated for visualization purposes.

The daily and seasonal models achieved better scores than the uniform distribution in 2021 as well as in 2020, although in this latter year the difference was slight (figure 7). The distribution of scores (in grey) for each model and year demonstrates the scores are smaller for the daily and seasonal models, despite ranges being equivalent to the uniform distribution in 2020. The daily model was slightly better than the seasonal model, in particular in 2021 (see the distribution of scores), but this difference was marginal. This result highlights that the daily and seasonal models achieved similar performances regarding the predicted number of individuals detected during the surveys.

## 4. Discussion

Our aim was to help fill the gap in our global understanding of the winter dynamics of cetacean distributions, which could have strong implications for the management of by-catches. This was achieved by, first, highlighting that the common dolphin distribution within the Northeast Atlantic shelves was related to environmental processes, with temporal delays specific to their nature and the cascading effects they had on the trophic chain. Second, our description of the infra-seasonal variations in common dolphin distribution sheds light on the poorly understood intrusions of the species within coastal waters during winter: the dolphins displayed a strong preference for the coastal-shelf waters front and extensively followed its spatial variations, with their overall distribution densities increasing over the period and peaking in March–April. This pattern was consistent across the two study years.

### 4.1. Methodological considerations

One of the limitations of this work might be the use of CDS rather than MCDS (multi-covariate distance sampling). For the sake of simplicity, we ignored the potential variations in detection according to the observation conditions, such as the effect of sea state. However, we filtered data with poor observation conditions (data were discarded when the sea state was higher than 3) to reduce this bias and we are confident about the robustness of the distance sampling procedures in such a context.

When fitting our models, we made certain choices to best capture the dynamics of the studied winters but which could prevent its direct use to predict the distribution of common dolphins in future winters. In particular, we used a forecast product of ocean conditions as the source for the environmental variables. This product comes as a 3 d forecast, i.e. the conditions are predicted for the 3 days ahead only. This implies that a model built with these variables might be used to predict 5–13 days in advance (depending on the shortest lags considered for the variables), if using only dynamic variables. This was not our case, however, since the best model to predict common dolphin distribution also includes the season-averaged distance to MLD front, which can only be computed once the complete winter period is over. As such, our model can only be used retrospectively, although with a short delay, but not for forecasting.

To assess the reliability of our predictions, we used a cross-simulation that took into account the study design. The classical cross-validation procedure, by randomly removing bits of the study design, impairs one of the basic prerequisites supporting distance sampling, i.e. the consistent coverage of the study area [49]. This degradation of the sampling design can result in overlooked and unpredictable consequences on the model quality, and our approach freed us from this bias. Yet, by being based on the realized survey effort, our approach comes with the limitation that we can only compare the predictive performance of models on those parts of the study area actually sampled. Yet, most of the divergence between our two models occurred outside the sampled areas and we have no way of assessing which model is more reliable in these places.

### 4.2. Delayed response to environmental conditions

We found results consistent with common dolphins preferentially associating with structures favouring water enrichment and prey availability, with a medium to long delayed (7–30 days) response to those structures. The MLD, SSH and salinity fronts worked best at a 10 d lag, which suggests they might be proxies more of water enrichment processes than of prey accumulation. The concentration in dissolved inorganic carbon (DissIC) worked best at the 10 d lag. Both Chl, NPP and Phyto stood out at the 7–10 d lag, which is consistent with expert knowledge on the reaction lag through the trophic chain in the area and their being a proxy of prey availability. The prey accumulation and prey accessibility variables came out in either short (as expected) or long (not as expected) lags. Distances to Chl and NPP fronts worked best at shorter lags (2–4 d lags), indicating they could be proxies of prey accessibility and accumulation for common dolphins. Conversely, distances to MLD and Temp fronts worked best at longer lags (30 days), suggesting they might rather be proxies for longer-term processes such as water enrichment. Euphotic depth (ZEu) and all MLD-related variables (MLD, gradMLD) explained better common dolphin distribution at medium lags, while we expected a shorter delay if these variables were proxies of aggregating features and of prey accessibility. Instead, this medium delay (7–10 d lag) is more indicative of those conditions being involved in enrichment features.

We expected a short time lag for Temp and Salinity if the common dolphin distribution was related to it through physiological effects. However, we found their distribution was better correlated to a long lag (10 days), opening the question as to what the effects of these parameters are on common dolphin distribution. Given the important variations in temperature and salinity between coastal and shelf waters, both conditions might be proxies of the closeness to shore and freshwater plumes, and in particular their extension towards the shelf. Common dolphins were associated with high-salinity areas, with a clear negative relationship with salinity, which is consistent with low salinity being physiologically less suitable for them [8]. Alternatively, common dolphin distribution might be indirectly related to these two parameters, through their influence on prey distribution.

Interestingly, the fronts of Chl, NPP and Temp, but also SSH and Current Speed, were all uninformative, suggesting none were proxies of any sort for common dolphin distribution in the study area. The same was true for the averaged distance to Temp front, as well as for Bathy. For the latter, this is probably due to the very low variability of bathymetric features in the study area, with the whole area being included in the known bathymetric preferences of common dolphins (50–200 m [15,23,64]). The same might stand for SSH, with a low variability of SSH spatial patterns across the study area over the course of the considered winter.

Conversely, the strong support for the persistence and seasonally averaged distance to MLD, Chl and NPP fronts emphasized the importance of recurrent frontal features in structuring the BoB ecosystem, by separating coastal and shelf waters [35,41].

Yet, there was low support for a relationship to short-term fronts, but a strong one to longer-term features might arise from the spatial scale considered in this work. Our models were built using 5 km segments, to best match the spatial resolution of the considered environmental conditions. Due to the hierarchical structure of the ocean [1], it could be that dolphins rely on ephemeral frontal structures at very fine scale (hours and meters, which is unobservable with the resolution of our data [65]), but at a larger scale, such as considered here (greater than 1 km), their distribution is related to longer-term features. Testing for this would necessitate even finer-resolution data, which would require a different observation platform to a plane (i.e. a small-sized boat) and to specifically target frontals areas.

### 4.3. Winter-scale variations in dolphin distribution

Our modelling approach allowed us to identify some of the main drivers of day-to-day variations in distribution, at a relatively small spatial scale, of the common dolphin in the Northeast Atlantic shelves. This work emphasized the importance of frontal areas in common dolphins’ ecology. Indeed, our results indicated they were preferentially observed in areas closest to the seasonally averaged MLD front, but also where the surface chlorophyl *a* concentration was intermediate. That is, the highest densities of common dolphins overlapped with the most productive areas of the shelf water mass, except in the highly productive coastal waters associated with the low-salinity river plumes. Those high chlorophyl concentrations were supported by water mixing (deep MLD); hence, the preference for both intermediate chlorophyl concentration at a 7 d lag and for deep MLD at a 10 d lag points towards common dolphins targeting areas where the water column was enriched through frontal mixing. The preference for intermediate distance to Chl fronts at a short time lag might suggest common dolphins fine-tune the preference for a frontal process at a lower spatio-temporal scale to find prey aggregation within the frontal area [65].

As a result, the common dolphin distribution seemed to follow the spatial variations of the coastal-shelf waters front, both in terms of localization and intensity, leading to several peaks of density throughout the winter. Those peaks were well supported by the data, with the lowest levels of extrapolation, and occurred mostly in March–April during the transition period from winter- to spring-time oceanographic conditions. The identified preference for frontal areas associated with river plumes seems to hold up to late spring (May–June), when the species exhibited an intermediate pattern between winter and summer distributions, with strong densities associated with plumes alongside strong densities over the shelf edge [10,18].

The predicted spatial dynamics of common dolphin distribution displayed variations between the two consecutive winters, mostly linked to the variations in oceanographic conditions in the frontal area. In 2020, common dolphins were mostly present within the northern part of the shelf, avoiding both coastal and outer shelf waters in January–February, but becoming more prevalent throughout the shelf in February–April, although still avoiding the coastal waters. In 2021, while a similar pattern occurred, common dolphins were more widespread, with density hotspots along the front, especially in March–April. This spatial dynamic and the predicted density peaks suggests that the coastal-shelf waters front attracted common dolphins from elsewhere during particular periods, either from offshore oceanic waters or from north- or southward shelves.

In the BoB, the first plankton blooms appear in late winter, during the transition from winter to spring in March–April [66–68]. This very first peak in zooplankton concentration, representing only a small fraction of the total seasonal production, might be driven by the adults emerging from diapause as well as from early recruitment from eggs produced by females at the end of winter (female *Calanus helgolandicus* are known to anticipate a phytoplankton bloom with their first egg production [66]). The 50 m isobath area of the south-half of the BoB is also known to be the main spawning area of anchovies in the area [69], where they start spawning in the southern part and then progressively migrate northward. In late winter, the coastal-shelf waters frontal area thus works as an aggregative feature for both emerging zooplankton and new recruitement, but also for ichtyoplankton (fish eggs mainly [70]) and spawning pelagic fishes, subsequently attracting predators (fish, seabirds, cetaceans). Therefore, the association of common dolphins with frontal areas highlighted by our results may be consistent with predators being attracted by the strong trophic activity associated with such discrete features.

This work adds a substantial new piece to the scattered knowledge about the seasonal variations in common dolphin distribution. Worldwide, winter surveys indicate that common dolphins move towards the continental shelves during the winter season, with peaks in density observed in the shelf waters in several areas in January–March (Gulf of Maine [25]; BoB [26,27,71,72]; Hauraki Gulf [14]; Madeira [29]). These studies unfortunately did not have sufficient resolution to further explore the mechanisms driving these coastal incursions, but we can hypothesize that similar mechanisms to the one described in our study area might be involved at a more global scale: during a season of oligotrophic offshore waters, common dolphins move towards coastal waters to target frontal zones sustaining strong prey productivity and aggregations. When winter gives way to spring and summer, the common dolphin distribution shifts back towards more oceanic waters [21]. In particular, common dolphins target the shelf break and its recurring, strong fronts supporting very high productivity (on both sides of the Atlantic, from the Hebridean shelf break to Gibraltar [15,22–24], as well as along the western Atlantic shelf edge [23,25,73]), but also specific features of production enhancement in an overall oligotrophic shelf. This is the case with the Celtic Sea fronts, for example, which are known to aggregate substantial densities of common dolphins throughout the summer [28,71,74,75].

The daily model performed only slightly better than the seasonal model in reproducing the number of common dolphins sighted per segment, implying a similar predictive performance. This result indicates that the seasonal model captured well the variations in common dolphin distribution. This was unexpected, since we hypothesized that common dolphins track small-scale processes, both spatially and temporally. Note, though, that the seasonal model was supported mostly by standard deviation variables, i.e. by the extent of variability in environmental conditions through the season, rather than by mean values. The same is true with the most important variable in the model, the averaged distance to MLD fronts, which captured the seasonal variation in day-to-day positions of the coastal-shelf waters front. The seasonal model therefore pointed towards the same direction as the daily model, by emphasizing the importance of infra-seasonal variability in environmental conditions and stressing the crucial significance of the coastal-shelf water front in common dolphin distribution.

Operationally, this result has substantial implications as it suggests that seasonal models may be equally useful as daily models for conservation management, at least if they encompass the contemporaneous seasonal variability. Indeed, we might expect that a seasonal model built on climatologies (i.e. conditions averaged over a multi-year period) would not achieve the same level of performance [16], as it would not capture the inter-annual and small-scale variability in strength and position of the coastal-shelf front actually used by common dolphins on a daily basis. Day-to-day models fitted to environmental parameters, as in this study, are being increasingly used as predictive tools for dynamic management of fisheries and their by-catch (see, for example, the EcoCatch tool [76]), but also to better inform conservation management of marine megafauna in relation to anthropogenic threats such as maritime traffic [77,78]. Here, we demonstrated that building daily models for common dolphin distribution is actually possible and informative, provided sufficient, recurring and independent fine-scale observation efforts [79]. The logical next step is to develop a predictive tool of the spatio-temporal distribution of common dolphins in the BoB at the winter scale. Such a tool would then allow finely tuned dynamic management decisions to be informed, potentially with flexible spatio-temporal closures or restrictions of fisheries [80,81], to reduce or prevent the high by-catch levels faced by this population [30,31].

## Data Availability

All files and codes used for the present analysis are available at https://www.seanoe.org/data/00756/86805/ [82].
